# Anatomical consideration of the cardiac plexus to prevent grave bradycardiac arrhythmias associated with lung cancer surgery: a case report

**DOI:** 10.1186/s40792-019-0686-6

**Published:** 2019-08-08

**Authors:** Kurumi Fukui, Shingo Ikeda, Toshiya Yokota, Tatsuhiro Hoshino

**Affiliations:** Surgical Department of Respiratory Center, Mitsui Memorial Hospital, 1 Kandaizumi-cho, Chiyoda-ku, Tokyo, 101-8643 Japan

**Keywords:** Lung cancer surgery, Arrhythmias, Bradycardia, Cardiac plexus, Vagus nerve

## Abstract

**Background:**

Arrhythmias are known as one of the complications of lung cancer surgery, and most of them are not lethal. Life-threatening arrhythmias have been reported in the literature but in reality very rare.

**Case presentation:**

A 67-year-old Japanese man with a history of hypertension was diagnosed with squamous cell carcinoma in left lower lobe underwent a left lower lobectomy and bilateral mediastinal lymph node dissection through a median sternotomy. During lymph node dissection along the right vagus nerve, the patient’s heart rate and blood pressure dropped suddenly and an electrocardiogram monitor showed ST elevation. These abnormalities returned to normal soon after cardiac massage was performed and a coronary vasodilator was given. A temporary pacing wire was inserted at the end of the surgery. The postoperative course was uneventful and the patient was discharged on postoperative day 11 without a need for permanent pacemaker.

**Conclusions:**

We present a patient who was complicated with lethal arrhythmias during lung cancer surgery for the purpose of elucidating, from anatomical viewpoint, the relationship between arrhythmias and the involvement of cardiac plexus during lymph node dissection. The result showed that arrhythmia was inadvertently elicited by cardiac plexus stimulation during lymph nodes dissection around the vagus nerve. It is important to be familiar not only with the course of phrenic, vagus, and recurrent laryngeal nerve but also the anatomy of cardiac plexus to prevent arrhythmic complications in lung cancer surgery.

## Introduction

Arrhythmias are known as one of the complications associated with lung cancer surgery, and most of them are not lethal. But life-threatening arrhythmias, such as asystole, have been described in the literature, but in reality very rare. We present a patient who was complicated with lethal arrhythmia during surgery. The purpose of this study is to elucidate from an anatomical viewpoint the relationship between arrhythmias and the involvement of cardiac plexus during lymph node dissection.

## Case presentation

**Fig. 1:**
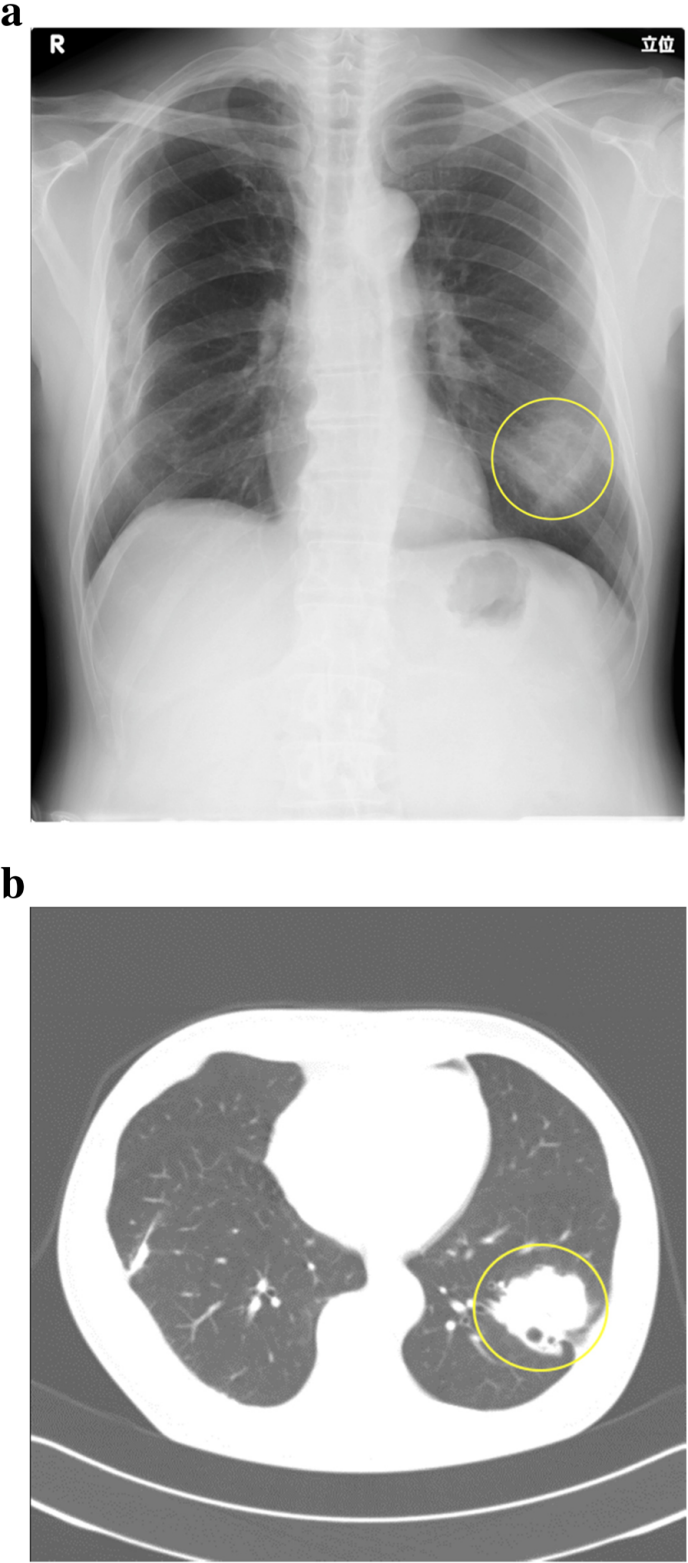
**a** Chest X-ray shows a 50-mm mass in the left lower field (yellow circle). **b** Chest CT scan shows a 47-mm tumor in the left lower lobe lateral basal segment (yellow circle)

**Fig. 2:**
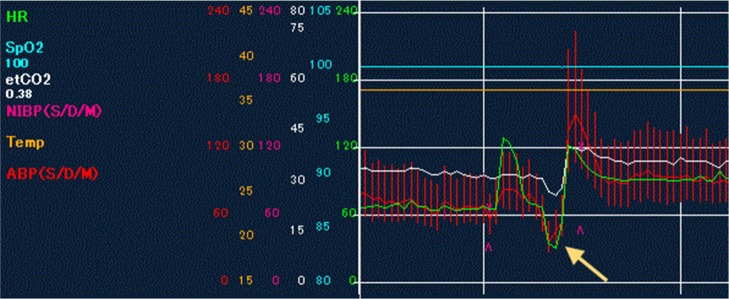
The monitor record shows the heart rate (green line and yellow arrow) dropped to 30s. It returned normal soon after a cardiac massage was performed and a coronary vasodilator was given

A 67-year-old Japanese man with a history of hypertension presented with bloody sputum. An X-ray, CT, and bronchoscopic biopsy led to the diagnosis of squamous cell carcinoma in left lower lobe, cT2bN0M0 stage IIA (Fig. 1a, b). A preoperative electrocardiogram showed a heart rate of 73 per minute with normal sinus rhythm and the echocardiography was normal. The patient underwent a left lower lobectomy and bilateral mediastinal lymph node dissection via median sternotomy. At first, lymph nodes in the bilateral superior mediastinum were dissected. During lymph node dissection along the right vagus nerve, the patient’s heart rate and blood pressure dropped suddenly (Fig. 2) and an electrocardiogram monitor showed ST elevation. These abnormalities returned to normal soon after a cardiac massage was performed and a coronary vasodilator was given. After the event, we safely finished the lymph node dissection around trachea and esophagus followed by bifurcation of the trachea. We added anterior axillary chest incision in the fifth intercostal space, and then dissected the lymph nodes around the aortic arch, with taping the left vagus nerve in order not to stimulate or damage it. We resected the left lower lobar artery, left lower lobe bronchus, then after dissecting the lymph nodes in the inferior mediastinum, the left lower lobar vein is processed and the specimens are removed. A temporary pacing wire was inserted at the end of the surgery. The postoperative course was uneventful and the patient was discharged on postoperative day 11 without a need for a permanent pacemaker.

## Discussion

In our institution, we have been performing bilateral mediastinal lymph node dissection via median sternotomy since 1987. Patients meeting the following criteria were considered for inclusion: (1) 75 years old or less, (2) performance status 0 or 1, (3) compatible cardio, respiratory, and renal function, (4) non-GGO, (5) solid nodule 2 cm or larger. We exclude small cell carcinoma, stage 4, and right lower lobe lung cancer. From January 1990 till December 2010, we performed 218 surgeries and arrhythmias occurred in 6 cases (2.8%), but the type of arrhythmias is unknown and we need to continue analyzing the data.

**Fig. 3:**
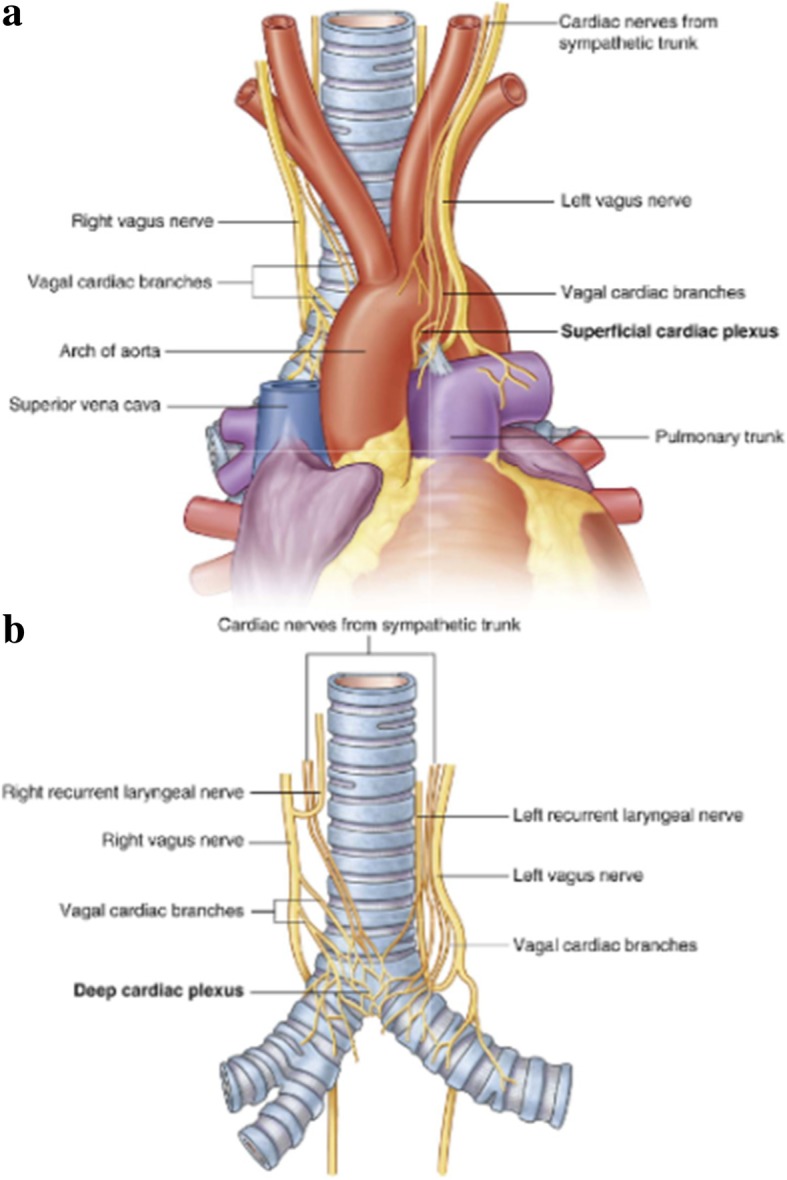
The anatomy of cardiac plexus, showing the superficial part (**a**) and the deep part (**b**) [1]

In lung cancer surgery, it is important to understand the anatomical courses of the vagus, phrenic, and recurrent laryngeal nerves. Among those nerves, the vagus nerve, especially the cardiac plexus, has a cardinal role in controlling the cardiovascular system. However, the construct of the cardiac plexus is complex enough to need some comments. The cardiac plexus is divided into the superficial and deep parts. The superficial part lies beneath the arch of the aorta, in front of the right pulmonary artery. It consists of cardiac branches from the left sympathetic trunk and the left vagus nerve. The deep part is situated in front of the bifurcation of the trachea, above the point of division of the pulmonary artery, and behind the aortic arch. It consists of small mixed branches from bilateral sympathetic and parasympathetic fibers that supply the heart. It is well established that these two parts are closely interconnected (Fig. 3) [2].

Some studies have shown that the cardiac function can be affected by electric stimulation of the vagus nerve as well as the sympathetic tone. Electrical stimulation of the left thoracic vagus nerve close to its cardiac branch resulted in a significant drop in the end-systolic elastance (Ees) of 38 ± 16% in human during open chest surgery for coronary artery bypass grafting. β-1 adrenoreceptor antagonism also reduced baseline Ees by a mean of 33 ± 11%, indicating a strong dependence of cardiac contractility on sympathetic tone under these conditions of anesthesia and open chest surgery [3]. Conversely, for the purpose of allowing critical placement of anastomotic sutures during a coronary artery bypass grafting, electrical stimulation of the left vagus nerve was utilized to slow down the heart rate and temporarily arrest the heart for a brief period of time [4].

Furthermore, direct physical stimulation to the vagus nerve also alters the cardiac function. Traction of the left vagal nerve in a patient operated on for a cervical cancer resulted in bradycardia and a blood pressure fall. A reflex response due to excitation of afferent vagal fibers was considered to be a culprit because the effects were eliminated when the vagal nerve trunk was blocked by xylocaine centrally to the site of the mechanical stimulation [5].

Therefore, either electrical stimulation or physical traction of the vagus nerve, or interception of the sympathetic system can decrease heart rate or even lead to a cardiac arrest.

In our department, we make it a rule to tract the vagus nerve using a soft rubber tape to avoid nerve damage during surgery. However, when we dissected the lymph nodes along the vagus nerve in the presented patients, the vagus nerve might have been stimulated physically or by an electronic device and resulted in significant bradycardia and asystole. In addition, epidural anesthesia using propofol may have depressed the sympathetic system and resulted in parasympathetic dominance, making the vagus nerve more likely to be stimulated.

## Conclusion

It is important to be aware that lethal arrhythmias might be triggered during lung cancer surgeries, especially in association with lymph node dissection in the vicinity of the vagus nerve. Vital signs should be carefully monitored throughout the surgery. Being familiar with the anatomy of cardiac plexus and avoiding physical and electronic stimulation might avoid grave arrhythmias associated with lung cancer surgery.

## Data Availability

Not applicable.

## Abbreviations

CPR: Cardiopulmonary resuscitation
CT: Computer tomography
GGO: Ground glass opacity

## References

[1] Drake R, Vogl W, Mitchell A (2015). Chapter 3: thorax. Gray’s anatomy for students.

[2] Gray H, Lewis W (1918). The great plexuses of the sympathetic system. Anatomy of the human body.

[3] Lewis ME, Al-Khalidi AH, Bonser RS, Clutton-Brock T, Morton D, Paterson D, Townend JN, Coote JH (2001). Vagus nerve stimulation decreases left ventricular contractility in vivo in the human and pig heart. J Physiol.

[4] Matheny RG, Shaar CJ (1997). Vagus nerve stimulation as a method to temporarily slow or arrest the heart. Ann Thorac Surg.

[5] Carlsten A, Folkow B, Hamberger C-A (1957). Cardiovascular effects of direct vagal stimulation in man. Acta Physiol Scand.

